# MeCP2 Is an Epigenetic Factor That Links DNA Methylation with Brain Metabolism

**DOI:** 10.3390/ijms24044218

**Published:** 2023-02-20

**Authors:** Yen My Vuu, Chris-Tiann Roberts, Mojgan Rastegar

**Affiliations:** Department of Biochemistry and Medical Genetics, Max Rady College of Medicine, Rady Faculty of Health Sciences, University of Manitoba, Winnipeg, MB R3E 0J9, Canada

**Keywords:** epigenetics, DNA methylation, MeCP2 isoforms, Rett Syndrome, glucose, cholesterol, brain metabolism, brain development, autophagy, BDNF, mTOR, AMPK

## Abstract

DNA methylation, one of the most well-studied epigenetic modifications, is involved in a wide spectrum of biological processes. Epigenetic mechanisms control cellular morphology and function. Such regulatory mechanisms involve histone modifications, chromatin remodeling, DNA methylation, non-coding regulatory RNA molecules, and RNA modifications. One of the most well-studied epigenetic modifications is DNA methylation that plays key roles in development, health, and disease. Our brain is probably the most complex part of our body, with a high level of DNA methylation. A key protein that binds to different types of methylated DNA in the brain is the methyl-CpG binding protein 2 (MeCP2). MeCP2 acts in a dose-dependent manner and its abnormally high or low expression level, deregulation, and/or genetic mutations lead to neurodevelopmental disorders and aberrant brain function. Recently, some of MeCP2-associated neurodevelopmental disorders have emerged as neurometabolic disorders, suggesting a role for MeCP2 in brain metabolism. Of note, *MECP2* loss-of-function mutation in Rett Syndrome is reported to cause impairment of glucose and cholesterol metabolism in human patients and/or mouse models of disease. The purpose of this review is to outline the metabolic abnormalities in MeCP2-associated neurodevelopmental disorders that currently have no available cure. We aim to provide an updated overview into the role of metabolic defects associated with MeCP2-mediated cellular function for consideration of future therapeutic strategies.

## 1. Introduction

Epigenetic mechanisms determine and dictate cellular morphology and function, without any change in the corresponding DNA sequences [1,2,3,4]. Such mechanisms include DNA methylation, RNA modification, three-dimensional chromatin structure, chromatin remodeling, nucleosome re-positioning, histone post-translational modifications (PTMs), and the activity of non-coding regulatory RNA molecules, among others [5,6,7]. Aberrant epigenetic mechanisms are associated with an extensive variety of human diseases, from different types of cancer, organ malfunction, developmental abnormalities, neurodevelopmental and neurological disorders, and mental disability [8,9,10,11]. Of note, DNA methylation has been extensively studied regarding normal physiological conditions of human health and disease states. One prominent example is methyl-CpG binding protein 2 (MeCP2), an epigenetic reader of DNA methylation, which was the first discovered member of the methyl-CpG binding protein (MBP) family. A wide range of neurodevelopmental disorders are associated with *MECP2* mutations/altered expression; including Rett Syndrome (RTT; loss-of-function), autism spectrum disorder (ASD; reduced expression), fetal alcohol spectrum disorders (FASD; altered expression), *MECP2* duplication syndrome (MDS; gain-of-function), and severe neonatal encephalopathy [12,13].

RTT is one of the most devastating neurodevelopmental disorders in females with an incidence of about 1:10,000 to 1:15,000 of live-born females [14,15]. Importantly, 95% of typical RTT and more than 50% of atypical RTT are caused by *de novo* mutations in the X-linked *MECP2* gene [16,17]. Clinical presentation of RTT begins from 6 to 18 months of age with several prominent characteristics; including stereotypical hand wringing, autistic behaviors, impaired verbal development, motor dysfunction, intellectual disability, seizures, breathing abnormalities, and digestion difficulties along with disturbed metabolism. One of the common indicators of RTT includes metabolic deficits, particularly in glucose and lipid/cholesterol metabolism. For instance, in patients with RTT as well as in RTT mouse models, multiple metabolic perturbations have been found, including insulin resistance, abnormal glucose levels, impaired lipid utilization, and aberrant mitochondrial function [18,19,20,21,22,23] (Table 1). Due to the suggested connection between MeCP2-associated diseases and metabolism, researchers have found certain cellular pathways (i.e., the mechanistic target of rapamycin (mTOR) complex and adenosine 5′-monophosphate activated protein kinase (AMPK) that may link MeCP2 functional properties to the brain metabolism.

Considering the potential of the metabolic drugs as treatment options for MeCP2-linked neurodevelopmental disorders, the focus of our review is to discuss the impacted cellular metabolism in MeCP2-associated diseases, focusing on RTT. This review will also provide supplementary mechanistic insight into these possible connections.

## 2. An Overview of DNA Methylation

### 2.1. History

Nearly a century ago, 5-methylcytosine (5mC) was first discovered in *Mycobacterium tuberculosis* [32], leading scientists to uncover a novel aspect of gene regulation. Twenty-five years later, DNA methylation at the 5C position of cytosine was confirmed in mammals, insects, and plants [33]. By 1968, researchers noticed that different levels of DNA methyltransferases (DNMTs) exist in different rat organs, suggesting that the overall 5mC content may vary between tissues [34]. It was then discovered that DNA methyltransferases are responsible for transferring the methyl groups from methyl donors to the fifth carbon of the cytosine nucleobases. Since its initial discovery, scientists have given more attention to DNA methylation, especially after the term “epigenetics” was first recorded in 1942 by Conrad Waddington [35]. Shortly thereafter, the primary role of DNA methylation was characterized in the bacterial immune system to be involved in DNA replication [36,37]. Thus, the long history of DNA methylation reflects not only the progressive discovery of evidence for this regulatory mechanism, but also the importance of investigations with a multidisciplinary approach in molecular biosciences.

### 2.2. DNA Methylation in the Central Nervous System (CNS)

Since the discovery of DNA methylation in the bacterial immune system and in DNA replication, epigenetic modifications have been thought to regulate gene expression and to contribute to genetic diversity via modulation of chromatin structure and chemical changes on DNA molecules and histones (DNA-bound proteins). Such chemical modifications would involve acetylation, methylation, phosphorylation, SUMOylation, ubiquitination, and ADP ribosylation [38]. Epigenetic mechanisms influence many aspects of human health, from development and homeostatic maintenance to pathophysiology of certain diseases. Amongst these, DNA methylation, along with other concomitant regulators, plays important roles in brain development and function.

It has been reported that DNA methylation plays significant roles in brain development and function, during embryonic development, extending into adulthood. For instance, after a zygote forms, a higher level of DNA methylation is found in the inner cell mass as compared to the trophectoderm. The inner cell mass progresses to embryonic formation with the development of the nervous system as the first step of embryogenesis, whereas the trophectoderm becomes the wall of the blastocyst. This suggests the critical role of DNA methylation in early stage of life. Moreover, the level of DNA methylation varies throughout different brain regions such as the cerebral cortex, cerebellum, and pons, implying their distinguished functions [39]. Furthermore, in cell type-specific processes, DNA methylation is involved in neurogenesis and gliogenesis [40,41]. In differentiated brain-derived embryonic neural stem cells from mice, DNA methylation, along with the transcript and protein levels of DNA methylation machinery (readers, writers, and erasers), is controlled in a sex- and strain-dependent manner [42,43]. The expression level of *Mecp2*/MeCP2 in primary murine neural stem cells, neurons, astrocytes, and specific brain regions is also controlled by DNA methylation at its regulatory regions (R): R1–R3 in the promoter and R4–R6 in the intron 1 [43,44,45,46,47]. In rats, DNA demethylation events take place in the seven cytosine–phosphate–guanine (CpG) di-nucleotides of the *Gfap* gene located far upstream of the promoter. This demethylation event particularly occurs between embryonic day (E) 14 to postnatal day (PND) 10, coinciding with the differentiation of astrocytes from multipotent neural progenitor cells (NPCs) [48]. *GFAP*/*Gfap* encodes the glial fibrillary acidic protein (GFAP) that uniquely contributes to the cytoskeletal dynamics of astrocytes. Moreover, DNA methylation facilitates hippocampal neurogenesis [48].

In post-mitotic neurons, abnormal level of DNA methylation can lead to aberrant synaptic plasticity and neuronal function. These aberrations were investigated in one study that applied the DNA methylation inhibitor 5-aza-2′-deoxycytidine (5AZA) in hippocampal cells of 21-day-old rats. The results showcased that altered level of DNA methylation led to dysfunctional hippocampal long-term potentiation (LTP) [49]. Similarly, another study proposed that introduction of contextual fear conditioning to adult rats altered DNMTs functions in the hippocampus, leading to decreased memory formation [50]. A later comprehensive assessment of DNA methylation profiles in 95,815 neurons and 8167 non-neuronal cells of mouse brain illustrated the apparent necessity of proper epigenetic regulation in the development of diverse structure and function of neurons in the cerebrum [51]. Thus, DNA methylation significantly contributes to brain development and function.

### 2.3. Different Factors Involved in DNA Methylation as an Epigenetic Modification

Typically, DNA methylation commences with covalent addition of a methyl group to the fifth carbon of a cytosine residue (generating 5mC)—a process catalyzed by DNA methyl transferases. Subsequently, readers of 5mC will take turns to control gene regulation through binding and interpreting these epigenetic modifications. Alternatively, erasers/modifiers of 5mC may further oxidize these epigenetic marks into other forms of DNA methylation (please see sections below).

#### 2.3.1. Writers of DNA Methylation

DNA methylation writers are enzymes of the DNMT family that includes DNMT1, DNMT2, DNMT3A, DNMT3B, and DNMT3L. These enzymes add methyl groups from S-adenyl methionine onto the fifth carbon of the cytosine residues to form 5mC. The DNMT1 enzyme is the maintenance DNMT that sustains the parental methylation pattern through the methylation of the hemi-methylated DNA molecules, while repairing DNA methylation errors [52,53]. Meanwhile, DNMT3A and DNMT3B function as *de novo* DNMTs and catalyze new methylation of naked DNA [54]. During mammalian embryonic development, DNMT3A is ubiquitously expressed after E10.5, but DNMT3B is mainly expressed in totipotent embryonic cells [55]. Thus, DNMT3B is crucially required for early cell division, proliferation, and differentiation after fertilization, prior to the action of DNMT3A. Studies have shown that knockout of *Dnmt1* and *Dnmt3b* in mice leads to embryonic lethality. Contrastingly, *Dnmt3a* knockout mice exhibited a survival rate that was still observed within the first four weeks of age [56,57]. These results show that DNMTs play significant roles during early development. DNMT3L, on the other hand, lacks the responsible domain for enzymatic activity but promotes the catalytic activity of DNMT3A/B as a transcriptional repressor [58,59]. DNMT2 is the most conserved member in this protein family and is involved in RNA methylation [60].

#### 2.3.2. Erasers/Modifiers of DNA Methylation

DNA demethylation is a process that may happen either passively or actively. While passive DNA demethylation is a non-enzymatic process that takes place in dividing cells after fertilization, active demethylation involves multiple enzymes. These events which occur post-fertilization prominently contribute to passive and active DNA demethylation with significant involvement in the maternal and paternal alleles [6]. In this regard, the paternal genome goes through active DNA demethylation before the onset of DNA replication through a series of enzymatic reactions. However, passive DNA demethylation occurs without enzymatic activity in the maternal alleles after cellular cleavage divisions [61]. During active DNA demethylation, the amine and methyl groups at the 5mC are chemically modified through several enzyme-catalyzed reactions. This includes the activation-induced cytidine deaminase (AID)/apolipoprotein B mRNA-editing enzyme complex (APOBEC), which catalyzes the deamination reaction converting 5mC to thymine. As a result, the base excision repair (BER) pathway is activated to modify the base. Furthermore, APOBEC promotes the ten–eleven translocation (TET) enzymes to oxidize the methyl group at the 5C position, forming 5-hydroxymethylcytosine (5hmC) [1,5,62,63,64]. In an in vitro study, it was shown that once 5hmC is formed, it can be switched back to cytosine through *de novo* DNMT catalyzation [65]. Studies have also shown that formation of 5-hydroxymethyluracil from the deamination of 5hmC can be further removed by a repair mechanism [5,66].

#### 2.3.3. Readers of DNA Methylation

After methyl groups are added onto the fifth carbon of the cytosines, DNA methylation readers can bind to these epigenetic modifications and interpret these epigenetic marks. This is accomplished by the action of MBPs which are categorized into three subgroups: the methyl-CpG-binding domain (MBD) family of proteins, the Set and RING-associated (SRA) domain-containing proteins, and the zinc-finger proteins. Proteins such as ubiquitin-like containing PHD ring finger domains 1 (UHRF1) and ubiquitin-like containing PHD ring finger domains 2 (UHRF2) contain the SRA domain that can bind to methylated DNA. Moreover, UHRF1 preferentially binds to hemi-5mC, while UHRF2 interacts with both hemi-5mC and hemi-5hmC [67,68]. Furthermore, zinc-finger proteins, including Kaiso, zinc finger and BTB domain-containing 4 (ZBTB4), and zinc finger and BTB domain-containing 38 (ZBTB38) can bind to methylated DNA and subsequently suppress transcription. Notably, ZBTB4 and ZBTB38 are highly expressed in the brain [69]. Amongst members in the MBP family, the MBD proteins were the first to be identified and characterized due to their relation to various cancers and brain disorders [70]. There are seven members in the MBD family: MeCP2, MBD1, MBD2, MBD3, MBD4, MBD5, and MBD6 [71]. As previously mentioned, MeCP2 is known for its critical contribution to the formation, function, and development of the brain.

### 2.4. MeCP2, a Well-Studied Epigenetic Reader of DNA Methylation

#### 2.4.1. MeCP2—Expression, Key Functional Domains, and DNA Binding Activities

**Figure 1:**
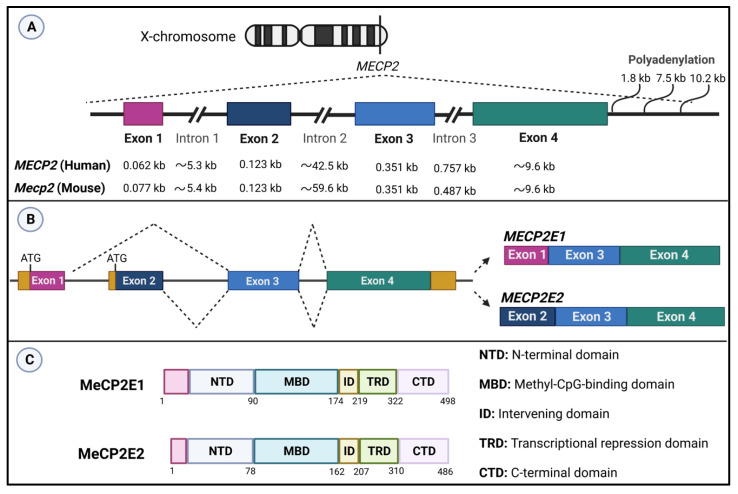
Schematic diagram of (**A**) *MECP2*/*Mecp2* gene, (**B**) *MECP2E1* and *MECP2E2* transcripts, (**C**) MeCP2E1 and MeCP2E2 isoforms. MeCP2: methyl-CpG binding protein 2. The dark yellow boxes show 5′ and 3′ untranslated regions (UTR). The drawing is not to scale. Information obtained from [12,74,75,77]. Figure is created with BioRender.com.

While MeCP2 is found throughout the body, its expression level is highly abundant in the brain compared to other organs. The high expression level of MeCP2 is observed in the postnatal brain [72] and in mature neurons [73]. MeCP2 is encoded by the X-linked *MECP2* gene (Xq28) that is composed of four exons and three introns. Alternative splicing at exon 1 and exon 2 creates two main isoforms called MeCP2E1 and MeCP2E2 [74,75] (Figure 1). These two MeCP2 protein isoforms have similar structure for all functional domains, except in their N-terminal domains. MeCP2E1 contains 21 unique amino acids in its N-terminus, while there are nine unique amino acids in the N-terminus of MeCP2E2. Earlier studies from our team have shown that MeCP2E1 and MeCP2E2 are expressed differently in distinct brain regions, although both are detected in neurons, astrocytes, and oligodendrocytes of young adult murine brain [44]. The two MeCP2E1 and MeCP2E2 isoforms show differential sensitivity to DNA methylation [46], and the murine *Mecp2* promoter can properly deliver their regulated expression in primary cortical neurons [76]. Additional coding and non-coding *MECP2* isoforms are predicted by in silico analysis of the publicly available databases [77]. MeCP2 protein contains five domains with specific functional properties. The N-terminal domain (NTD) modulates methyl-CpG-binding domain activity and turnover rate of MeCP2 [78]. Although the differences in MeCP2E1 and MeCP2E2 isoforms are minimal, their disease contribution varies, with MeCP2E1 mutation being sufficient to cause RTT-associated symptoms, and MeCP2E2 being dispensable for RTT. The MBD is responsible for binding to methylated CpG di-nucleotides, while the intervening domain (ID) helps in stabilizing the structure and binding capacity of MBD [79,80]. The transcriptional repression domain (TRD) mediates transcriptional repression via recruitment of the corepressor complexes, and the C-terminal domain (CTD) is suggested to facilitate MeCP2 in binding to chromatin [81,82]. Different MeCP2 protein domains are shown in Figure 1.

The discovery of MeCP2 binding to methylated DNA was initially reported in the 5mC-enriched sequences that are located in pericentromeric heterochromatin regions and chromocenters [75]. Recently, it has been noted that in the murine brain, MeCP2 is dynamically present in the liquid-like form of chromatin condensates. In this regard, the functional contribution of MBD and the C-terminal of the intrinsically disordered regions of MeCP2 are critically involved in these chromatin condensates [83]. Although the main DNA binding capacity of MeCP2 is detected in methyl CpG-rich pericentromeric genomic regions, MeCP2 can also bind to methylated non-CpG sequences. A study in the murine brain showed that methyl CAC sequences harbor high affinity towards MeCP2 through MBD compared to methyl CAA, methyl CAG, methyl CAT; a similar trend was also found in hydroxymethylation of the same sequences [84,85]. Moreover, MeCP2 can recognize methylated and hydroxymethylated CpA di-nucleotides, mediated by MBD [86]. The variation in MeCP2 binding capacity highlights the diverse roles of MeCP2 in brain development and functional properties of mature neurons.

#### 2.4.2. Mechanism of Action of MeCP2 in Transcriptional Regulation

**Figure 2:**
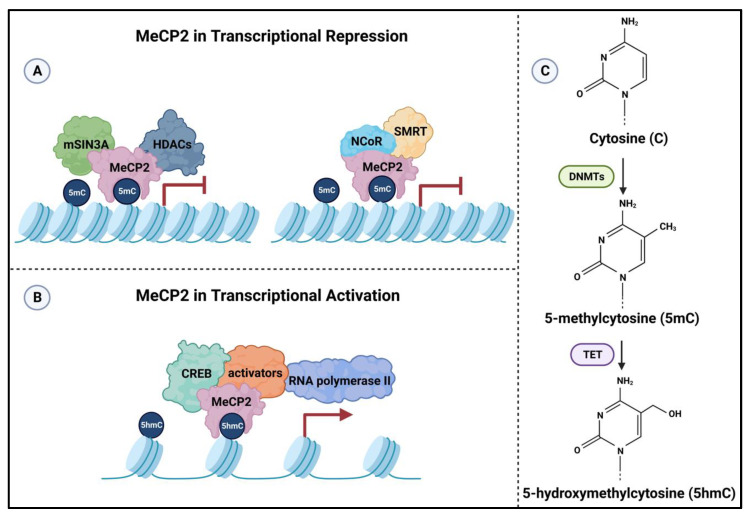
The role of MeCP2 in transcriptional regulation. (**A**) Upon binding to methylated CpG di-nucleotides (5mC), MeCP2 recruits repressor complexes such as mSIN3A, HDACs, and NCoR-SMRT to suppress gene transcription. (**B**) Upon binding to 5hmC, MeCP2 interacts with CREB to promote transcriptional activation and recruitment of other activators. (**C**) DNMTs add methyl groups onto the fifth carbon of cytosine residues in the CpG di-nucleotides to form 5mC, whereas TET enzymes oxidize the methyl groups to form 5hmC. 5hmC: 5-hydroxymethylcytosine; 5mC: 5-methylcytosine; CREB: cAMP response element binding protein; DNMTs: DNA methyltransferases; HDACs: histone deacetylases; mSIN3A: mammalian switch-independent 3A; MeCP2: methyl-CpG binding protein 2; NCoR: nuclear receptor corepressor; SMRT: silencing mediator for retinoid and thyroid hormone receptors; TET: ten–eleven translocation. Information obtained from [13,15,53,54,64,87,92,93,94]. Figure is created with BioRender.com.

In terms of transcriptional regulation, MeCP2 is known to mediate both activation and suppression of gene expression (Figure 2). This duality in its functional roles is mainly implemented through the activity of its two functional domains: MBD and TRD. MeCP2 is a nuclear protein that binds to methylated CpG di-nucleotides but can also bind to non-methylated DNA. Upon binding, the TRD of MeCP2 interacts with transcriptional suppressive complexes such as mammalian switch-independent 3A (mSIN3A) and histone deacetylases (HDACs) [87]. HDAC1 and HDAC2, which belong to class I of the HDAC family, are engaged in MeCP2-linked transcriptional regulation [88]. The transcriptional repression activity of MeCP2 is also known in the context of lentiviral silencing in embryonic stem cells [89]. The basic structure of chromatin involves nucleosome structures with an octamer of histones (2 histone H3, 2 histone H4, and 2 dimers of H2A-H2B) with 146 base pairs of DNA molecules wrapped around them. Normally, acetylation of lysine residues of histone tails help neutralizing their positive charge, which may reduce the interactions between the positive charges of lysine residues with negative charges of phosphate groups present in DNA sequences. This results in a relaxation of the chromatin structure, allowing the DNA molecules to become more available for the binding of transcriptional factors. On the other hand, HDACs catalyze the removal of acetyl groups from lysine residues located at the N-terminus of histone tails, causing chromatin compaction and reducing its accessibility. Thus, the collaboration between MeCP2 and SIN3A-HDAC is crucial to regulate gene expression, which in turn contributes to normal physiological function of the body, especially in the brain. Studies have demonstrated the importance of HDAC1 and HDAC2 in prenatal development as well as the essential connection between HDAC1, HDAC2, and MeCP2 in synaptic transmission [90,91]. Moreover, disturbance of MeCP2-linked transcriptional regulation may lead to abnormal behaviors. For instance, a study using conditional *Hdac1* and *Hdac2* knockout mice displayed abnormal grooming behavior. The authors noted a dysfunction in the SAP90/PSD-95-associated protein 3 (SAPAP3) in the striatum that was associated with excessive grooming in the mice. Consequently, the results of this study indicated that the expression level of SAPAP3, which is a member of the postsynaptic family of proteins that are involved in excitatory synapses, required the successful interaction between MeCP2 and HDAC1/HDAC2. Accordingly, these results suggest that HDAC1/HDAC2 recruitment through MeCP2 is vital in moderating repetitive behaviors [88].

Besides the interaction with mSIN3A/HDACs, MeCP2 also collaborates with the multiprotein complex NCoR/SMRT to repress transcription. The interaction between MeCP2 and NCoR/SMRT was observed through a specific missense mutation in *Mecp2* which created a point mutation, R306C, at the TRD. The authors showed that the R306C knock-in mice displayed severe behavioral defects, reduced brain weight, and premature death. At the protein level, there was no detection of the NCoR/SMRT complex in the brain nuclei of *Mecp2*^R306^ 5-week-old mice compared to the wild type. This insinuates a possible elimination of the interaction between MeCP2 and NCoR/SMRT via TRD [92]. Subsequently, co-repressor nuclear receptor corepressor (NCoR) and the silencing mediator for retinoid and thyroid hormone receptors (SMRT) both recruit other transcriptional suppressors such as HDAC3, G protein pathway suppressor 2 (GPS2), and the transducing β-like factors [95,96,97,98,99]. Recently, it has been proposed that transcriptional repression by MeCP2 on highly methylated DNA for long genes (>100 kilobases) requires the interaction between MeCP2 and NCoR repressor complex. In this regard, MeCP2 binding to methylated DNA resulted in a reduced rate of RNA polymerase II at the transcription start sites, which may lead to suppressed transcriptional initiation [100]. Of note, MeCP2 homeostasis regulation is through a regulatory network that involves BDNF-MeCP2 regulation [101,102,103,104,105]. The regulatory link of BDNF-MeCP2, in part, involves MeCP2 phosphorylation at specific serine residues that contribute in response to certain drugs [106,107].

Furthermore, MeCP2 may also function in transcriptional activation of its target genes. In such cases, for example, at the *Sst* gene, researchers noted the cohabitation of cAMP response element binding protein (CREB) 1 (a transcriptional activator) and MeCP2 on the promoter of *Sst*. In contrast, CREB1 activity was downregulated at the promoter of *Mef2c*, which is transcriptionally inhibited by MeCP2 [93]. Upon the successful interaction between MeCP2 and CREB1 on the promoter of its target genes, other co-activators are recruited to enhance transcriptional activation. One prominent example is histone acetyl transferase, which acetylates histone proteins, such as histone H3 lysine 27 (H3K27), by relocating the acetyl group from acetyl-CoA molecules to lysine residues of histone tails. This acetylation results in the relaxation of chromatin structure, thus promoting transcriptional activation. Similar transcriptional activation was also reported in neurons, where high level of 5hmC was detected. One study proposed the interaction between MeCP2 and 5hmC through MBD with a high affinity similar to that of 5mC. This proposed interaction suggests that while MeCP2 binding to 5mC may lead to transcriptional suppression, its binding to 5hmC may result in transcriptional activation of certain genes in CNS [94]. Moreover, the release of MeCP2 through phosphorylation on S421 mediated by the calcium/calmodulin-dependent protein kinase II (CaMKII) pathway can lead to e gene activation [108].

#### 2.4.3. MeCP2 and Regulation of Gene Expression in the Central Nervous System

Since *MECP2* gene mutation was discovered to be the underlying cause of RTT [16,17] and its association with other neurodevelopmental disorders such as Angelman syndrome, FASD, MDS, and ASD [109], a connection was made evident between MeCP2 and brain function. Particularly, it has been shown that MeCP2 is a key protein contributing to the development of the CNS with roles in neuronal maturation, dendritic morphology, neurite formation, as well as synaptic plasticity and transmission [13,110,111].

Moreover, mouse models have previously highlighted these MeCP2-facilitated developmental milestones. For example, it was reported that mild expression of MeCP2 begins on E10.5. However, at E11.5, a greater level of MeCP2 is found in the spinal cord, pons, and medulla. Then, at E14.5, MeCP2 is present in the thalamus, caudate putamen, and cerebellum. In mice, at E16.5, MeCP2 is detected in the deeper layers of the cerebral cortex and cerebellar nuclei as well as in the hypothalamus and hippocampus. From PND0 to 28 weeks old, the MeCP2 level is relatively uniform in the whole brain lysates [112]. In addition, many findings proposed the importance of MeCP2 in certain brain regions and in a cell type-specific manner. For instance, while MeCP2 expression is not only widely distributed during cerebellar development [113], selective differences in levels of MeCP2 expression between neurons and astrocytes exist [47,73]. Remarkably, the two main isoforms of MeCP2, MeCP2E1 and MeCP2E2, are also differentially expressed in distinct brain regions. While the level of MeCP2E1 is uniform in the olfactory bulb, striatum, cortex, hippocampus, thalamus, brainstem, and cerebellum, MeCP2E2 is highly expressed in the cerebellum, olfactory bulb, hippocampus, and striatum [44]. Additionally, we recently reported that MeCP2 activity on major cellular signaling pathways are dose-dependent, providing some insight into its neurobiological contribution in RTT and MDS [103].

Overall, MeCP2 harbors a robust role in brain function and progression towards neural developmental milestones, at the beginning of neuronal differentiation, and in mature neurons. Furthermore, MeCP2 is differentially expressed in a region-dependent manner and is cell type-specific-dependent in both human and murine brain/brain cells [42,43,44,45,46,47,73,101,102,104,114]. Given that MeCP2 participates in regulating dynamic gene expression, researchers have highlighted its vital involvement in neurobehavioral abnormalities that are linked to many complications such as metabolic impairments. Therefore, by gaining further insight into its role in regulating brain development, a greater understanding of MeCP2-linked neurological diseases can be unveiled in near future.

#### 2.4.4. Impaired Metabolism in Mutated MeCP2-Linked Neurodevelopmental Disorders

The abnormal alterations of metabolism are regularly seen in mutated MeCP2-associated diseases. Mutations in the *MECP2* gene/or its altered expression cause an extensive variety of human diseases ranging from neurodevelopmental disorders to neurodegenerative diseases: RTT, ASD, FASD, X-linked mental retardation, severe neonatal encephalopathy, MDS, Angelman syndrome, PPM-X syndrome, Huntington disease, early onset schizophrenia, and Hirschsprung’s disease [16,109,115]. Among these, most neurodevelopmental disorders are associated with metabolic impairments (summarized in Table 1).

Common metabolic abnormalities include glucose and cholesterol levels along with dampened mitochondrial function in both the circulatory and nervous systems. For example, studies have highlighted such metabolic impairments in RTT that is associated with mutated MeCP2 protein [17]. Insulin resistance was previously reported in RTT patients, while other studies have demonstrated aberrant lipid (especially cholesterol) and carbohydrate metabolism. Moreover, studies have shown that the activity of mitochondrial oxidative phosphorylation had diminished in two cases of severe neonatal encephalopathy. Similarly, altered glucose metabolism and abnormal levels of lipids were observed in other findings related to ASD and FASD (Table 1). Both glucose and cholesterol are critical to normal brain development, physiology, and function. Importantly, glucose does not only act as a main source of energy, but it also aids in neurotransmitter homeostasis [116]. Similarly, cholesterol harbors vital roles such as contributing to cell membrane integrity, signal transduction, as well as aiding in the formation of myelin, dendrites, and synaptosomes [117,118,119,120]. Thus, alleviating symptoms of mutated MeCP2-associated neurodevelopmental disorders may also require supplementary studies of the accompanying metabolic defects.

## 3. Brain Metabolism

**Figure 3:**
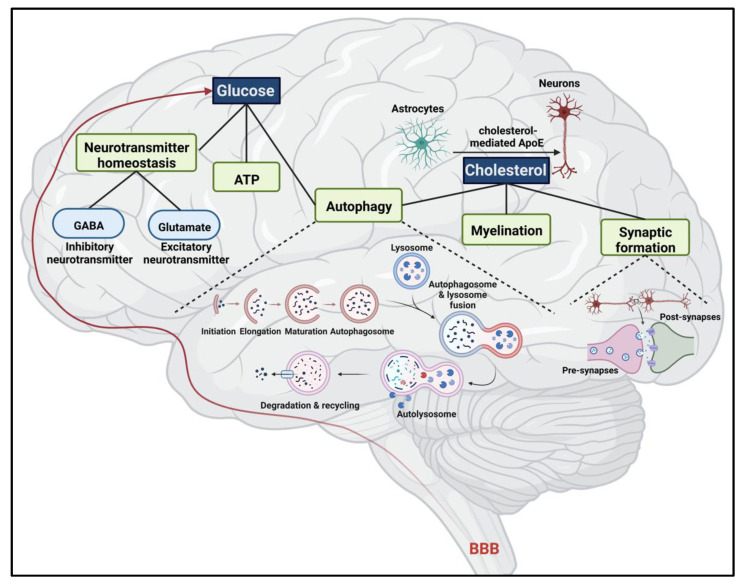
Key roles of glucose and cholesterol in the brain. Glucose enters the brain through the blood-brain barrier (BBB) and is subsequently involved in ATP production, neurotransmitter homeostasis and autophagy activity. In the adult brain, cholesterol is mainly synthesized in the astrocytes and then delivered to neurons by transporter “ApoE”. In the brain, cholesterol greatly contributes to myelination, synaptic formation, and regulates autophagy. ApoE: apolipoprotein B; ATP: adenosine triphosphate; BBB: blood-brain barrier. GABA: gamma amino butyric acid. Information obtained from [119,121,122,123,124,125,126,127,128,129,130,131,132]. Figure is created with BioRender.com.

Dynamic metabolic activity, particularly involving glucose and cholesterol, is important for the maintenance of biological function in the CNS. These two essential components are involved in different aspects of energy and neurotransmitter production, cell survival rate, and homeostasis from infancy to adulthood (Figure 3).

### 3.1. Glucose

Adequate amount of glucose is essential for proper brain function. Glucose is an important substrate in the brain, not only serving as the primary energy source but also as a key component in many critical pathways such as autophagy and neurotransmitter homeostasis. For instance, neurons are exclusively dependent on glucose for energy generation, along with the utilization of certain metabolites from its metabolism to produce neurotransmitters such as gamma amino butyric acid (GABA) and acetylcholine in the soma. Both GABA and acetylcholine are important neurotransmitters that facilitate communication between the brain and other parts of the body.

#### 3.1.1. ATP Production

One major role of glucose metabolism in the brain is to provide energy (Figure 3). In mammals, about 50% of adenosine triphosphate (ATP) production from glucose oxidation occurs in the mitochondria and is stably observed in the brain during an anesthetic state. It has been believed that this amount of energy is used to maintain transmembrane Na^+^/K^+^ ion gradients, implying that the brain has a large demand for glucose at any given state [121]. While the brain mass accounts for about 2% of our total body weight, it consumes close to 20% of the total glucose. In other words, for every 100 g of brain tissues, approximately 5.6 milligrams of glucose may be utilized per minute [133]. Relaying information between brain regions and distinct cell types relies on glucose-derived energy in the brain. As such, cortical signaling pathways heavily depend on mitochondrial glucose oxidation, while non-signaling processes require about 20% of the available energy support [134]. Importantly, the brain consistently requires glucose from the bloodstream due to insufficient glycogen storage in astrocytes [135]. This is further highlighted by local glucose-derived energy in neurons, which determines neuronal properties, including neuronal plasticity that thoroughly relies on aerobic glucose oxidation. This suggests that brain cells are sensitive to glucose limitation [136]. Therefore, abnormal glucose levels in the brain may cause serious neurological consequences.

The threat to normal neurological physiology posed by abnormal glucose levels was underscored in a case study related to a serious hypoglycemia-induced neurological issue that was reported in Belgium in 2012. In this case study, a 35-year-old patient was mechanically ventilated in an intensive care unit due to detection of *Escherichia coli* in the peritoneal fluid after gastroenterostomy (creating a direct connection between the stomach and the jejunum) alongside other disease conditions such as type 2 diabetes, depression, and hypothyroidism. On the 8th day, the patient underwent a series of unexpected complications, including severe high blood pressure, acute intracranial process, and deepened sedation. More importantly, the blood glucose level was reported as 0.0 mg/dL, as a report of blood gas readout. However, after intravenously introducing 12 g of glucose, the status of the patient greatly improved. Two weeks later, the patient was discharged without any sign of neurological problem, followed by a 6-month check-up [137]. This case study denoted how glycemic control is essential to the brain function, as prolonged and severe hypoglycemia can potentially lead to a life-threatening condition.

#### 3.1.2. Neurotransmitter Homeostasis

Glucose plays an important role in neurotransmitter homeostasis. Through glucose metabolism, astrocytes release “glutamine” [122,123], which is then converted into glutamate and GABA in neuronal cells, the chief neurotransmitters in the central nervous system [138] (Figure 3). Here, a glucose molecule is converted into two pyruvates via the glycolytic pathway in the cytoplasm. Then, pyruvate is converted to acetyl-CoA, which then enters the tricarboxylic cycle (TCA) in the mitochondrial matrix. Through several reactions in TCA, α-ketoglutarate (α-KG) is formed as an intermediate biomolecule. Subsequently, α-KG molecules exit the TCA cycle to enter the glutamine–glutamate–gamma amino butyric acid cycle [139]. Consequently, glutamate (an excitatory neurotransmitter) and GABA (an inhibitor neurotransmitter) are both formed from glutamine, which itself is originated from glucose metabolism.

Another neurotransmitter that is synthesized from glucose metabolites is “acetylcholine”. The two primary substrates in acetylcholine synthesis are acetyl-coenzyme A (acetyl-CoA) and choline; both substrates participate in a reaction that is catalyzed by the enzyme choline acetyltransferase. During acetylcholine synthesis, choline is absorbed into the terminals of pre-synaptic cells through a sodium-dependent glucose transporter [140], while acetyl-CoA is made available as a metabolite from glucose, lipid, and amino acid metabolism. Acetylcholine is primarily involved in the neuromuscular junction and in muscular contraction [141]. Further, acetylcholine mainly functions within the parasympathetic nervous system [142], aiding the body in relaxation after a stress response. In the brain, the source of acetyl-CoA metabolite may originate from either glycolysis, beta-oxidation of long-chain fatty acids in the cytosol, or amino acid oxidation. However, the brain primarily prefers to synthesize acetyl-CoA from glucose via the glycolytic pathway. This preference for the glycolytic pathway is due to the fact that fatty acid oxidation requires significantly more oxygen than glucose. For example, 1 mol of glucose requires 6 mol of O_2_ for an oxidation reaction, while palmitic acid requires 23 mol of O_2_ [143,144]. Another disadvantage of utilizing fatty acids as a glucose resource is that there is an increased production of superoxide (a radical of reactive oxygen species (ROS)) during beta-oxidation, which may cause immense oxidative stress in brain cells [144]. Hence, the preference of glucose in the brain for energy production as well as metabolites for neurotransmitter synthesis is garnered from greater oxygen use efficiency and reduced risk of ROS-induced brain damage.

#### 3.1.3. Glucose Metabolism in the Brain and the Link with Autophagy

Autophagy is a degradative process of dispensable cellular components with critical inputs to cellular metabolism. In the starvation state, cellular metabolism homeostasis is preserved through autophagic recycling of energy and nutrients that is supplied to cells for their survival [145]. It is proposed, however, that autophagy can be negatively affected by either glucose deprivation, an abnormal, elevated level of glucose, or insulin resistance, potentially leading to neuronal impairment. Specifically, one study examined whether autophagy could rescue neuronal cell death after a period of glucose deprivation, followed by glucose reintroduction into the cortical neurons derived from rat embryos. The results demonstrated that autophagic factors such as microtubule-associated protein light chain 3B-II (LC3B-II) (a marker for autophagosome formation) and P62/SQSTM1 (a predictor of autophagic flux) were significantly increased during the first 2-h time frame of glucose deprivation. Notably, when glucose was reintroduced into the cells, LC3B-II and P62/SQSTM1 levels were significantly reduced. However, despite a glucose replenishment period of 12–24 h following glucose deprivation, cortical cell death persisted, ultimately peaking at 60% of neuronal death after 24 h. Inhibition of autophagy had rescued cell viability [124]. This finding implies that an abnormal stimulation of autophagy, through disturbance of glucose metabolism, such as hypoglycemia, in the brain can lead to sustained neuronal cell death.

Conversely, hyperglycemic conditions may also impair autophagy in the brain. A high level of glucose in the hippocampus (a brain region notably associated with learning and memory) has been suggested to cause reduced autophagy and decreased autophagic flux. In one study, tissues of 30-week-old Goto-Kakizaki rats with hyperglycemia were used to examine the connection between autophagy machinery and neurotoxicity. Here, the level of LC3B-II was greatly reduced in the hyperglycemic condition, paralleled with an elevated level of P62/SQSTM1. This aberrant autophagy correlated to neurotoxicity and neuronal cell death in the hippocampus. This suggests that cellular protection, which is normally facilitated by autophagy, was restrained by abnormally high glucose levels [125]. Therefore, hyperglycemic conditions in hippocampal neurons may lead to suppressed protective autophagy and impaired cellular homeostasis. As a result, there appears to be a strong connection between glucose level and autophagy that helps to maintain homeostatic regulation of neuronal function (Figure 3).

### 3.2. Cholesterol

The brain is a unique organ regarding cholesterol metabolism. It contains the highest level of cholesterol compared to other organs, accounting for approximately 23% of total cholesterol in the body [146]. In the CNS, disturbances in cholesterol levels may lead to negative outcomes, such as altered biological function of cellular membranes. For instance, studies have shown that an abnormally reduced level of cholesterol in the brain may lead to amyloidogenesis (amyloid deposits) at the neuronal membranes in the hippocampus of patients with Alzheimer’s disease [147]. Additionally, the role of cholesterol has also emerged in other features of the brain such as myelin formation and synaptic transmission. Given the tight regulation of the cholesterol pool normally found in the brain, impairment of cholesterol metabolism may result in neurological disorders.

#### 3.2.1. Cholesterol Homeostatic Mechanism in the Brain

Dietary cholesterol and cholesterol made in the liver cannot cross the blood-brain barrier (BBB) to enter the brain. Consequently, the brain must synthesize cholesterol on its own. Astrocytes and neurons are the two brain cell types, where cholesterol is synthesized. During development, both neurons and astrocytes synthesize and release cholesterol. However, neurons produce a significantly higher amount of cholesterol than in astrocytes to support the process of myelination facilitated by oligodendrocytes [148,149]. Of note, cholesterol synthesis is mainly performed in astrocytes in the adult brain. Neuronal supply of cholesterol is thus maintained primarily by apolipoprotein E (ApoE), which transfers cholesterol from astrocytes to neurons [126] (Figure 3).

To prevent accumulation in the brain, cholesterol in neurons is converted to a metabolite called 24S-hydroxycholesterol (an oxysterol) that can cross the BBB. This process is catalyzed by the enzyme cholesterol-24 hydroxylase, encoded by the CYP46A1 gene [150]. In this regard, cholesterol-24 hydroxylase is responsible for the turnover rate of cholesterol in the brain, as it possesses around 80% of the total 24S-hydroxycholesterol found in the body [151]. In addition, production of 24S-hydroxycholesterol can facilitate the supply of cholesterol from astrocytes to neurons by signaling the former to increase cholesterol production [127]. The conversion of cholesterol to 24S-hydroxycholesterol has two critical purposes. First, as mentioned earlier, 24S-hydroxycholesterol from neurons signals to astrocytes to increase their cholesterol synthesis. Second, the excess level of neuronal 24S-hydroxycholesterol will be dispersed across the BBB to prevent cholesterol accumulation. Overall, the maintenance of cholesterol levels in the mature brain is tightly regulated by both astrocytes and neurons, whereby the former is the primary producer, and the latter is responsible for the highly efficient turnover rate.

#### 3.2.2. Major Role of Cholesterol in Myelin and Synaptic Formation

Most of the cholesterol in the human nervous system is found in the myelin sheaths (about 80%) [152], which are formed by oligodendrocytes in the CNS and by Schwan cells in the peripheral nervous system (PNS). Myelin is a protective layer of nerve axons, functioning as an insulator and conductor of electric impulses. This insulation allows the electric signals between nerve cells to be conducted at efficient speeds. Considering the high proportion of cholesterol in the CNS myelin (approximately 40.1%) [128], myelin cannot be formed in the absence of cholesterol. The necessity of cholesterol in myelin formation is highlighted by a study that involved the removal of exons 4 and 5 of the Fdft1 gene that encodes squalene synthase, an enzyme in the cholesterol synthetic pathway, in mice. The resulting enzymatic inactivation led to significantly delayed myelin formation in the brain and spinal cord. Although mice survived, they developed severe phenotypes such as ataxia and tremors [119]. Simultaneously, the PNS in this mutant mouse model showed great hypomyelination of the sciatic nerve, causing peripheral neuropathy and hindlimb weakness [153]. This finding proposed that cholesterol is indispensable and linked to the rate of myelin formation in CNS and PNS (Figure 3).

In addition, cholesterol is important in forming synapses and dendrites (Figure 3). In one study, purified rat retinal ganglion cells (RGCs) were introduced into media with the same concentration of cholesterol (treatment) and into glia-conditioned media (GCM) as control. The results showed that cholesterol completely mimicked the biological characteristics of GCM. Although cholesterol-induced synapses were smaller than synapses in GCM-derived medium condition, the number of synapses under cholesterol treatment was significantly higher [130]. Thus, these results suggest that cholesterol is essential for synaptic development in the nervous system. In another study, the researchers denoted that hippocampal synaptogenesis required intrinsically produced estradiol (a potent estrogen derived from cholesterol) as an essential component [129].

Besides synaptogenesis, studies also revealed the role of cholesterol in synaptic transmission. Cholesterol facilitated the conformational change of vesicle associated soluble N-ethylmaleimide-sensitive factor attachment protein receptors (v-SNARE) transmembrane domain that switched an open scissor-like structure to a close scissor-like shape. This change facilitates the fusion of synaptic vesicles into the cellular membrane to release neurotransmitters [154]. Other groups of scientists have also suggested the critical role of cholesterol in SNARE-mediated trafficking and exocytosis [155]. Thus, cholesterol is crucial to the development of myelin during brain development as well as in synaptic formation and transmission. These cholesterol-dependent functions help optimize the communication both between different brain regions and with other parts of the body.

#### 3.2.3. Cholesterol and Autophagy

A correlation between cholesterol and the autophagy pathway has been noted in many studies. For example, in 2018, Colell and colleagues used a mouse model of Alzheimer’s disease to determine the effect of cholesterol accumulation on autophagic activity. The mice expressed abnormally higher rates of cholesterol synthesis as well as cholesterol circulation. In addition, the increase in cholesterol on the lysosomal membranes disturbed the fusion of the autophagosome and lysosome (a digestive enzyme-containing organelle), impairing autophagy. This led to the abnormal accumulation of amyloid β protein (Aβ) [131]. Similarly, another group utilized PND7 *Npc1*^−/−^ heterozygous mice to examine the correlation of cholesterol accumulation and autophagy in the brain. A loss of functional Niemann—Pick C1 protein 1 (NPC1) protein interfered with the transfer of LDL-derived cholesterol from lysosomes to sub-organelles for reutilization. However, the results showed that with allopregnanolone (a GABA-positive allosteric regulator) intervention, abnormal cholesterol levels had reduced, followed by the amelioration of autophagy/lysosomal activity [156].

Furthermore, impairment of cholesterol metabolism in the brain is associated with neurological diseases such as Huntington’s disease (HD), Niemann–Pick type disease (NPC), and spinocerebellar ataxia (SCA) [132,157,158]. One research group found a potentially viable treatment for SCA by studying the link between cholesterol and autophagy. The SCA3 mouse model was introduced to adeno-associated viral vectors encoding CYP46A1 to improve the turnover rate of cholesterol in the brain. The results displayed an improvement in cholesterol turnover correlated with the recovery of autophagy activity [132]. Normally, the balance of the cholesterol pool in the brain is maintained via a key enzyme, CYP46A1. Thus, this study suggested that a dysfunctional CYP46A1 may lead to an abnormal accumulation of cholesterol in the brain. Consequently, cholesterol buildup may impair lysosomal function, hindering autophagosome–lysosome fusion. As a result, the autophagy pathway is impaired in the brain—a phenomenon often associated with neurological diseases. Therefore, the dynamic between abnormal cholesterol levels in the brain and impaired autophagic activity is implied to have prominent roles in many neurological diseases. Therefore, studying the relationship between neuronal cholesterol regulation and brain development is important, not only for possible therapeutic targets, but also for understanding the role of nutrient metabolism in the pathophysiology of certain neurological disorders (Figure 3).

## 4. The Interplay between MeCP2 and Brain Metabolism

**Figure 4:**
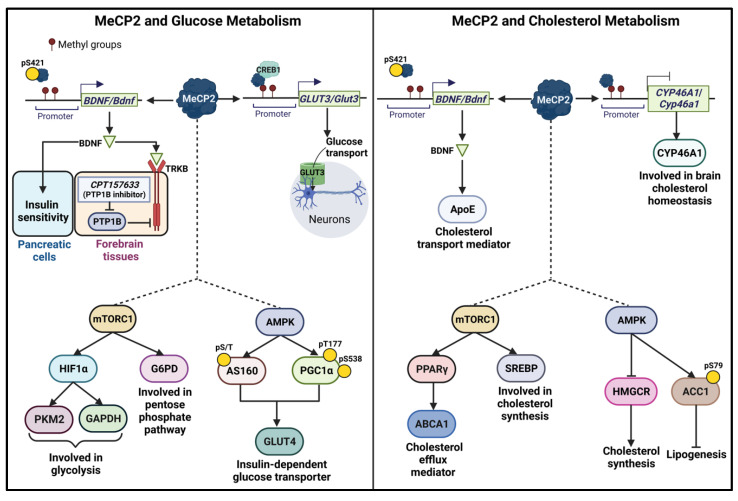
The potential links between MeCP2 with glucose and cholesterol metabolism. Brain-derived neurotrophic factor (BDNF), a downstream target of MeCP2, is engaged in both glucose and cholesterol metabolism; similar to mTORC1 and AMPK signaling pathways. Moreover, MeCP2 may regulate the expression of CYP46A1, which is a key factor in brain cholesterol homeostasis. MeCP2 promotes expression of GLUT3, which is a crucial transporter of glucose to neurons. ABCA1: ATP binding cassette subfamily A member 1; ACC1: acetyl-CoA carboxylase 1; AMPK: adenosine 5′-monophosphate activated protein kinase; AS160: Akt substrate of 160 kDa; BDNF: brain-derived neurotrophic factor; CREB1: cAMP response element-binding protein 1; CYP46A1: cytochrome P450 family 46 subfamily A member 1; G6PD: glucose-6-phosphate dehydrogenase; GAPDH: glyceraldehyde-3-phosphate dehydrogenase; GLUT3: glucose transporter 3; GLUT4: glucose transporter 4; HIF1α: hypoxia-inducible factor 1-alpha; HMGCR: 3-hydroxy-3-methyl-glutaryl-CoA reductase; MeCP2: methyl-CpG Binding Protein 2, mTORC1: mechanistic target of rapamycin complex 1; PGC-1α: peroxisome-proliferator-activated receptor gamma coactivator 1 alpha; PKM2: pyruvate kinase M2; PPARγ: peroxisome proliferator-activated receptor gamma; PTP1B: protein tyrosine phosphatase 1B; SREBP: sterol regulatory element-binding protein, TRKB: tropomyosin receptor kinase B. Information obtained from [18,150,159,160,161,162,163,164,165,166,167,168,169,170,171,172,173,174,175,176,177,178,179,180,181,182,183]. Please note that the molecular signaling cascades showing the link between MeCP2 and glucose metabolism as well as cholesterol metabolism were taken from different organs and cell type-specific regulation. Figure is created with BioRender.com.

As mentioned earlier, about 95% of RTT cases stem from a wide range of mutations in the MECP2 gene. Importantly, one of the common indications of RTT includes metabolic deficits, particularly in the glucose and lipid/cholesterol metabolism. Thus, MeCP2 is a strong candidate to help illustrate the interplay between DNA methylation readers and brain metabolism (Figure 4).

### 4.1. MeCP2 and Glucose Metabolism

Impairment of glucose metabolism has been closely linked to altered neural activity in RTT patients. In 2002, a study reported an increased rate of glucose metabolism in the cerebellum of RTT patients between the ages of 3 to 15, as well as in the frontal regions of the brain in patients 3 to 8 years old [22]. Notably, previous studies have highlighted similar trends in cerebellar glucose utilization in patients with autism as compared to healthy individuals [184]. Considering that autistic behavior is common in RTT patients, it suggests that disturbed neuronal glucose metabolism could depict a side component of other neurodevelopment disorders associated with MeCP2 mutations/altered expression. Contrastingly, a mildly lower rate of glucose metabolism was found in the lateral occipital areas in RTT patients [22]. This mildly lower rate of glucose metabolism was also explored in one study using *Mecp2* null mice that demonstrated how impairment of enzymes such as voltage-dependent anion channel 3 (VDAC3) (abundant in the outer mitochondria membrane), cytochrome C oxidase subunit 5a (COX5a) (complex IV), NADH dehydrogenase-ubiquinone 1 beta subcomplex subunit 8 (NDUFB8) (Complex I), and ATP synthase F1 subunit β (ATP5B) (Complex V) in the respiratory complexes of neuronal mitochondria, resulted in significant redox imbalance [24]. Additionally, an in vitro study demonstrated that cultured retinal neurons from adult goats in a hyperglycemic condition (25 mM of glucose) compared to a normal condition (5 mM) for 10 days, grew with abnormal length [185]. A recent study proposed significantly increased expression of MeCP2 and HDAC1 in streptozotocin-induced hyperglycemic mice, suggesting a strong connection between glucose metabolism and MeCP2 [186]. Hence, abnormal glucose metabolism may act differently in cell type- and brain-specific manners and may worsen the symptoms in RTT patients.

Similarly, the patterns of glucose and insulin intolerance were also noted. One study reported that in RTT mice (male *Mecp2*^−/y^ and female *Mecp2*^−/+^), there was an increase in blood glucose, coupled with insulin resistance and a lower rate of glucose absorption as compared to control mice. Notably, these mice also exhibited reduced level of insulin-induced tyrosine phosphorylation of IR-β and IRS1, leading to negative regulation of the downstream PI3K/AKT signaling pathway [168]. This is important, as PI3K/AKT signaling regulates and promotes glucose and lipid metabolism as well as cellular growth and survival [187]. In addition to abnormal glucose metabolism and insulin resistance, it is also important to consider other factors related to MeCP2 that may affect glucose metabolism in RTT. Therefore, elucidating the role of MeCP2 in glucose metabolism is critical during early intervention to address glucose metabolic abnormalities in patients with RTT and other MeCP2-associated neurological disorders.

#### 4.1.1. Glucose Transporters

Studies with transgenic mice have reported that MeCP2 is recruited to facilitate the transcription of solute carrier family 2 member 3 (*SLC2A3*/*Slc2a3*), also known as *GLUT3*/*Glut3*, encoding the glucose transporter 3 (GLUT3) protein. Researchers noted a methylation frequency at the CpG-rich sequences in the 5′-flanking regions of *Glut3* at PND14 that was significantly higher than PND3. The study also revealed that DNMT3A catalyzes the *de novo* methylation of CpG di-nucleotides on *Glut3* while also working succinctly with recruited MeCP2 and CREB1 to upregulate *GLUT3* expression of [159] (Figure 4). While studies had shown that the levels of both GLUT1 and GLUT3 were heightened in the prenatal brain, it was only the elevated level of GLUT3 in the postnatal brain that was associated with neuronal maturation [188]. Considering that GLUT3 has highest glucose affinity, amongst neural glucose transporters (GLUTs) [189], the uptake of glucose to neurons considerably supported by GLUT3 is implied. These findings suggest that the interplay between MeCP2 and neural glucose uptake harbors potential significance in disease development and/or progression.

There are other glucose transporters that are critical for transporting glucose. The brain consistently requires glucose from the bloodstream due to an insufficient glycogen storage in astrocytes, which normally has enough glucose to only last about 16 min [135]. Blood glucose, mainly from dietary carbohydrates and gluconeogenesis occurring in the liver and kidneys, enters the brain through the blood-brain barrier. Then, glucose molecules are absorbed into brain cells to produce ATP. While GLUT1 transporter is found in astrocytes [190], GLUT2 functions as a glucose sensor in the hypothalamus, involved in the regulation of daily food intake [191]. Moreover, GLUT5 serves as a glucose/fructose transporter in microglia [192,193]. In general, GLUT1, GLUT2, and GLUT5 are widely distributed insulin-independent transporters. In contrast, GLUT4 and GLUT8 are insulin-dependent transporters that specifically contribute to early blastocyst development and adult hippocampal memory formation [194,195,196]. It is suggested that there is a correlation between GLUT4 expression and epigenetic changes, particularly in the mechanism of DNA methylation that is found in the promoter of *Glut4* gene [197].

Abnormal MeCP2 expression can lead to disturbance of glucose uptake, resulting in the lack of a main source of energy for neurons. Accordingly, this may lead to a cascade of metabolic impairments that is evidently shown in patients with RTT.

#### 4.1.2. Brain-Derived Neurotrophic Factor (BDNF) Involvement in Glucose Homeostasis

*BDNF* is a direct target gene of MeCP2 [160,176], and its impaired level is reported in RTT [102]. BDNF plays vital roles in promoting the differentiation of progenitors, synaptic transmission, axonal branching, and dendritic growth [198,199]. Furthermore, it is known that BDNF regulates brain glucose metabolism in mouse models [161]. Considering the interplay between aberrant neural glucose metabolism and RTT pathophysiology, it is vital to explore the role that BDNF plays in MeCP2-linked neurodevelopmental disorders.

In 2015, Tonks and colleagues observed elevated protein tyrosine phosphatase 1B (PTP1B) protein levels in *Mecp2*-mutant mice (male *Mecp2*^−/y^ and female *Mecp2*^−/+^) [168]. PTPB1 is a negative regulator of insulin signaling pathways [200]. Thus, increased PTPB1 levels may induce insulin resistance. Tonks and colleagues also investigated the correlation between PTP1B, BDNF, and tyrosine receptor kinase B (TRKB; BDNF receptor) signaling. Upon treatment of CPT157633 (a PTP1B inhibitor, with 5 mg/kg of body weight) in RTT mice, homeostatic BDNF levels had been rescued—a result that correlated with an increase in TRKB phosphorylation [168] (Figure 4).

Other findings also suggested the growing association between BDNF and glucose homeostasis both in the brain and the rest of the body. For example, one study showed that a single loss-of-function mutation in BDNF within the hypothalamus of an 8-year-old female patient resulted in hyperphagia (excessive appetite), obesity, impaired cognitive function, and memory [201]. This finding suggested a hypothalamic circuit involving BDNF signaling that governs brain glucose homeostasis. Another study further describes the robust role of BDNF in glucose metabolism by proposing a possible role for BDNF-linked insulin secretion by pancreatic β-cells [202] (Figure 4).

Overall, it is logical to conclude that BDNF may present an important mechanistic link between *MECP2* mutations and RTT-related symptoms. In particular, in terms of glucose metabolism, observed phenotypes such as insulin resistance, abnormal glucose metabolism in the brain, and high blood glucose could be partially attributed to the interplay between MeCP2 and BDNF within RTT pathophysiology.

#### 4.1.3. mTOR, a Principal Regulator of Glucose Metabolism

The anti-fungal antibiotic rapamycin, found from Easter Island soil, derives its name from its mechanistic target of the rapamycin complex (mTOR) [203,204]. This protein is derived from the phosphoinositide 3-kinase (PI3K)-related kinase family, indicating that mTOR acts as a kinase that phosphorylates certain proteins, particularly on hydroxyl groups of serine and threonine. There are two main subset complexes of mTOR, namely mTORC1 and mTORC2, with different impacts in cellular metabolism. Specifically, mTORC1 is involved in several pathways such as ATP-dependent pathways and the SREBP pathway, which help regulate glucose uptake, metabolic homeostasis, and cell growth [205,206]. Notably, there is growing evidence showing the association between MeCP2 and both mTORC1 and mTORC2 complexes [177,178,179,180]. In this regard, mTORC1 potentially regulates signaling cascades that are linked with glucose metabolism.

It has been noted that glycolysis is upregulated through the PI3K/AKT/mTORC1 cascade as a result of the Warburg effect. Simultaneously, researchers noted that mTORC1 activates hypoxia-inducible factor 1-alpha (HIF1α) [164,167]. Separate studies have since corroborated the role of HIF1α in glycolysis by highlighting the induction of enzymes pyruvate kinase M2 (PKM2), which is a pyruvate kinase (PK) isoform, and glyceraldehyde-3-phosphate dehydrogenase (GAPDH) [166,167] (Figure 4). The upregulation of PK through PI3K/AKT/mTORC1 is particularly important, as it catalyzes the conversion of phosphoenolpyruvate to pyruvate, the final product of glycolysis, after which pyruvate converts to acetyl-CoA, which then enters to mitochondria for adenosine triphosphate (ATP) production, the body’s main energy-carrier molecule, via the tricarboxylic acid (TCA) cycle and electron transport chain (ETC).

The mTORC1 signaling pathway also leads to the stimulation of the pentose phosphate pathway (PPP) via SREBP/G6PD (Figure 4). It has been proposed that impaired mTORC1 level causes downregulation of sterol regulatory element-binding proteins (SREBP) and glucose-6-phosphate dehydrogenase (G6PD), resulting in a blockage of PPP [165]. G6PD is a rate-limiting enzyme in PPP and catalyzes the conversion of glucose-6-phosphate (G6Pase) to 6-phosphogluconate. PPP is very important to cellular biological function, as it not only provides resources for DNA replication, but also produces NADPH, which aids in the maintenance of redox homeostasis. Meanwhile, the mTORC2 complex facilitates glucose metabolism via AKT-mediated glucose uptake in brown adipose tissues to maintain thermal homeostasis [207]. Additionally, mTORC2 regulates hepatic glucose metabolism through processes such as insulin-mediated glucose uptake, gluconeogenesis, and glycolysis. These processes are facilitated via the insulin/AKT/mTORC2 signaling pathway [208,209].

Remarkably, components of both mTORC1 and mTORC2 complexes are impaired in the brain of RTT patients [177], which was similarly found in mouse models with mutations in *Mecp2* [178]. Overall, the evidence suggests that mTOR complexes may play a mechanistic role between MeCP2 and disturbed glucose metabolism.

#### 4.1.4. AMPK: A Key Regulator in Energy Homeostasis

Glucose metabolism is also regulated by the adenosine 5′-monophosphate activated protein kinase (AMPK) pathway. In an in vitro study using human primary skeletal muscle cells, AMPK phosphorylates histone deacetylase (HDAC) 5, a transcriptional repressor, at serine 259/498, which restrains the suppressive effect of HDAC5 on *GLUT4* transcription. Thus, the researchers suggested that the AMPK/HDAC5/GLUT4 pathway may be a potential therapeutic pathway in treating diabetes and insulin resistance [210]. A similar observation was likewise reported in terms of the AMPK/PGC-1α/GLUT4 signaling pathway. In vitro and in vivo studies illustrated that AMPK acted as a kinase of peroxisome-proliferator-activated receptor gamma coactivator 1 alpha (PGC-1α) at threonine 177 and serine 538 to stimulate PGC-1α. The activated PGC-1α promotes the expression of GLUT4, particularly in skeletal muscles [163] (Figure 4). Moreover, studies from another group suggested that nitric oxide may be a significant effector of AMPK that stimulates AMPK activity, leading to increased expression of GLUT4 [211]. Similarly, the AMPK/Akt substrate of the 160 kDa (AS160) signaling pathway also stimulates the translocation of GLUT4 in response of elevated blood glucose and insulin secretion [162] (Figure 4).

The effect of abnormal AMPK signaling in MeCP2-linked diseases was recently examined in Mecp2 null mice. The metabolic fingerprinting results had detected elevated AMP and ADP levels. This may lead to an observed increase in AMPK-mediated activation of glycolysis and TCA [181,212]. Hence, it is believed that impaired function of MeCP2 can disturb the AMPK signaling pathway and that it results in several symptoms in RTT patients, including high blood glucose and insulin resistance.

### 4.2. MeCP2 and Cholesterol Metabolism

Similar to glucose metabolism, abnormal cholesterol metabolism is also found in RTT patients, RTT mouse models, and other MeCP2-associated neurodevelopmental disorders. Both systemic and brain cholesterol homeostasis is disturbed in RTT pathophysiology. This perturbation of cholesterol homeostasis was demonstrated in *Mecp2* null mice, as an aberrantly elevated level of cholesterol was found in the liver and in the brain. The resulting downstream effects involved a significant downregulation of cholesterol synthesis as negative feedback [18]. Further, dysregulated cholesterol homeostasis was depicted in RTT patients having a noticeably higher level of low-density lipoprotein (LDL) along with an elevation in LDL receptor (LDLr) protein levels by 60% as compared to healthy individuals [213]. This indicates an RTT-specific disruption of cholesterol clearing from the bloodstream. In contrast, in RTT, the level of scavenger receptor class B type I (SR-BI), which acts as a receptor for high-density lipoprotein (HDL) and is responsible for removing excessive cholesterol from the peripheral tissues, is reduced [214]. Additionally, RTT patients exhibited altered levels of cholesterol metabolism-related proteins in the bloodstream. For example, compared to healthy individuals, a noticeable reduction of 3-hydroxy-3-methyl-glutaryl-CoA reductase (HMGCR) level was observed in RTT donor-derived fibroblasts. HMGCR is an enzyme involved in cholesterol synthesis. In addition, the level of LDLr, which allows for uptake of cholesterol into cells, was greatly increased in RTT patients. These cholesterol metabolism-related indicators showed a considerable alteration in cholesterol homeostasis in RTT. In the same study, the level of SREBP-2, which is involved in the synthesis of cholesterol and fatty acids, was significantly elevated [213]. In a study using mouse models exhibiting RTT-like phenotypes, cholesterol synthesis was partially repressed in the brains of mature RTT male mice compared to controls [215]. Hence, disturbed cholesterol metabolism is a strong component of RTT development.

#### 4.2.1. Involvement of BDNF in Cholesterol Homeostasis in the Brain

BDNF contributes to cholesterol homeostasis and metabolism in the brain. It is a downstream target of *MECP2* that becomes deregulated in RTT [216]. Normally, BDNF promotes the synthesis of cholesterol in cultured neurons of the cortex and hippocampus of 20-day-embryonic rats, which then contributes to the collection of pre-synaptic vesicles and synaptic integrity [217]. In one study using the human neuroblastoma cell line SH-SY5Y and the human glioblastoma–astrocytoma cell line U-87, recombinant ApoE3 and BDNF proteins were introduced into cells. Their results showed that BDNF controls ApoE (apolipoprotein E) synthesis and subsequently delivers the synthesized cholesterol in the astrocytes into neurons (Figure 3). This was illustrated in a dose-dependent manner, whereby increasing the level of BDNF (5, 10, and 20 ng/mL) resulted in an increase in cholesterol efflux (15%, 35%, and 44%, respectively) compared to controls (Figure 4). Alternative benefits elicited by BDNF were also indicated by a reduction of cholesterol-induced apoptotic activity (programmed cell death) in the neuroblastoma cell line SH-SY5Y [169]. Therefore, impaired BDNF levels can cause disturbed cholesterol homeostasis, while negatively impacting synaptic development.

#### 4.2.2. mTOR: A Principal Regulator of Cholesterol-Related Cellular Pathways

Researchers have suggested that mTORC1 mediates the localization of lipin 1, a Mg^2+^-dependent phosphatidic acid phosphatase, which subsequently regulates the activity of SREBP. As a transcription factor, SREBP targets genes involved in lipogenesis by binding to the sterol regulatory elements of DNA sequences, particularly in different brain regions where de novo cholesterol synthesis occurs [218,219,220,221] (Figure 4). In the mammalian brain, cholesterol is locally synthesized in neurons and glial cells during development. Upon reaching maturity, the overall yield of cholesterol synthesis in neurons is reduced. This reduction infers that cholesterol homeostasis in the brain depends on cholesterol synthesis in the astrocytes (a type of specialized glial cell) [222].

When inactivating mTORC1, lipin 1 enters the nucleus to downregulate SREBP. Consequently, the process of lipids/cholesterol synthesis is inhibited [174]. mTORC1 may activate the transcription factor peroxisome proliferator-activated receptor (PPAR)γ, which then promotes the efflux of cholesterol. In normal cellular conditions, PPARγ acts as a ligand as it binds to its nuclear receptor to upregulate the expression of ATP-binding cassette subfamily A member 1 (ABCA1). ABCA1 evokes cholesterol efflux from macrophages [175] (Figure 4). As it relates to RTT, a recent study showed that dysregulated mTOR levels may negatively affect certain genes that encode particular transcription factors (SREBP, SP1, and NF-Y) involved in sterol/cholesterol synthesis in the developing brain [223]. SP1 and NF-Y are factors that contribute to the production of 7-dehydrocholesterol reductase [224], which catalyzes the conversion of 7-dehydrocholesterol to cholesterol. Hence, dysregulated mTOR pathway may cause impaired cholesterol metabolism, resulting in impaired early brain development.

#### 4.2.3. PI3K-PKB/Akt Signaling Pathway: A Companion to Cholesterol Metabolism

Since the discovery of AKT family of proteins in 1977 [225], characteristics of the respective pathway(s) have been well-studied. Along with primary contributions to cellular proliferation, survival, and biological homeostasis, the AKT pathway is also involved in metabolism through PI3K-PKB/Akt signaling pathway. It is reported that when AKT, also known as protein kinase B, was activated through the stimulation of secondary messenger phosphoinositide 3-kinases (PI3K), it induced the activity of the SREBP protein. Consequently, expression of enzymes involved in fatty acids and cholesterol synthesis such as fatty acid synthase (FAS) and HMGCR was elevated [226]. AKT is also linked to mTORC1. This link is highlighted by AKT phosphorylating the proline-rich AKT substrate of 40 kDa (PRAS40) to dissociate the binding of PRAS40 from mTORC1. This dissociation event leads to the release of activated sites on mTORC1, followed by mTORC1-mediated regulation of SREBP. For instance, when insulin binds to its receptor, there is initiation of the AKT/mTORC1 signaling cascade whereby insulin stimulates PI3K, leading to AKT activation and subsequent dissociation of PRAS40-mTORC1 complex [227,228]. Moreover, the link between AKT and mTOR pathway provides a powerful cascade for promotion of myelination. Thus, PI3K/Akt/mTOR pathway acts as a feedforward control to produce a large pool of cholesterol during brain development in order to optimize myelinogenesis and axon ensheathment [149]. A recent study from our team also demonstrated that the AKT pathway acts in an MeCP2 dose-dependent manner in the human brain and/or brain cells [103].

#### 4.2.4. Cholesterol-24 Hydroxylase: A Critical Enzyme in Brain Cholesterol Homeostasis

Cholesterol metabolism in the brain is notably dissimilar to that of systemic circulation. This disparity exists since the brain requires localized synthesis of cholesterol in neurons and astrocytes. The brain strictly regulates the balance of cholesterol content through crosstalk between neurons and astrocytes. This interaction is vital, especially during development. While both neurons and astrocytes synthesize and release cholesterol, neurons produce significantly more cholesterol to support myelin production by oligodendrocytes. However, in the adult brain, cholesterol synthesis is largely inhibited in neurons and is primarily produced by astrocytes. If there is a lack of cholesterol in neurons, ApoE facilitates the transportation of cholesterol from astrocytes to neurons. To prevent accumulation of cholesterol in the brain, cholesterol in neurons is converted to a metabolite called 24-hydroxycholesterol, in a reaction catalyzed by an enzyme called cholesterol-24 hydroxylase (CYP46A1, cytochrome P450 46A1), encoded by the CYP46A1 gene, to cross the blood-brain barrier for excretion. Moreover, 24-hydroxycholesterol from neurons may target and initiate signaling in astrocytes to trigger cholesterol synthesis to support neurons. It is proposed that when 24-hydroxycholesterol from neurons enters the astrocytes, it enhances the expression of ApoE protein through a liver X receptor (LXR)-controlled pathway [127]. However, manipulation of cholesterol metabolism by the LXR-controlled pathway was likewise observed in systemic circulation [229]. As a result, altered levels of CYP46A1 may lead to cholesterol accumulation in the brain, which then negatively impacts cholesterol synthesis and other cholesterol metabolites. In vivo models also corroborate this dynamic, as the level of *Cyp46a1* transcripts in 28-day-old *Mecp2* null mice was increased approximately 40% as compared to wild type mice. However, at PND56, the level of *Cyp46a1* was downregulated in mice with mutated MeCP2 [18]. On the other hand, another study showed no significant differences of *yp46a1* mRNA levels at any specific age (PND14, 43, 56) [215]. The potential outcome of *Cyp46a1* transcript levels may help drive attention to the brain cholesterol levels before and at the onset of RTT and other MeCP2-linked brain disorders as well as to the link between MeCP2 and expression of the genes involved in cholesterol metabolism (Figure 4). Despite these inconsistencies, however, it is apparent that a fluctuating or abnormal level of cholesterol in neural development can lead to brain dysfunction due to the important role of cholesterol in postnatal brain physiology.

#### 4.2.5. AMPK: A Critical Regulator in Cholesterol Metabolism

AMPK is not only a key factor in maintaining energy homeostasis, but it also plays a role in cellular lipid/cholesterol balance. Homeostatic conditions normally involve AMPK inhibition of lipogenesis and de novo cholesterol synthesis via AMPK-induced phosphorylation of acetyl-CoA carboxylase 1 (ACC1), which suppresses enzymatic activity [170]. In the cytoplasm, ACC1 normally catalyzes the conversion of acetyl-CoA to malonyl-CoA, which is then converted into fatty acids through a series of reactions. Moreover, AMPK diminishes SREBP activity. In mammals, transcription factor SREBP(s) regulates the homeostatic system of cholesterol and fatty acid synthesis by activating the release of their precursors from the membranes of endoplasmic reticulum so that these biomolecules are available for cholesterol and fatty acid synthesis [172]. AMPK deactivates HMGCR via phosphorylation and inhibits the conversion of HMG-CoA to mevalonate [171]. Overall, AMPK harbors a critical role in neural cholesterol metabolism, making it a prime target for statins (which are cholesterol-lowering drugs) [230,231]. Given the wide scope of metabolic pathways that AMPK is involved in, and the vital role of these metabolic pathways, particularly in early neural development, further exploration of mechanistic relations between AMPK and MeCP2 becomes increasingly vital.

### 4.3. Advancements in Research Targeting MeCP2 in Neuronal Pathologies Involving the Link between Brain Metabolism and DNA Methylation

MeCP2 is a well-studied epigenetic factor that functions as the main reader of DNA methylation in the brain. While its expression is found throughout the body, it is most abundant in the brain. Hence, *MECP2* mutations and/or altered expression are largely associated with neurodevelopmental and neurological disorders [109]; many of these disorders are linked to metabolic impairments (Table 1). Although a direct link between MeCP2 and brain metabolism remains to be fully explored, emerging studies that focus on DNA methylation/MeCP2 and metabolism in the context of neuronal pathologies provide insight about this relationship. Accordingly, commonly used drugs that are administered in patients to control glucose and cholesterol metabolism are subjects of study for MeCP2-associated disorders such as Rett Syndrome and other diseases of the brain such as brain tumors [18,20,104,232,233,234,235].

Apolipoprotein E (ApoE) is the principal cholesterol mediator in the human body, especially in the brain. The ApoE protein is polymorphic, including ApoE2, ApoE3, and ApoE4, which are differentiated through distinct amino acids at positions 112 and 158. Cysteine is present at positions 112 and 158 in ApoE2, while ApoE4 has arginine at both positions. ApoE3 has a distinct arginine only at position 158 [236,237]. Amongst the three members of APOE family, ApoE4, encoded by *APOEε4*, has been considered to become a potential modulation factor for the RTT phenotype. This finding showed a significant difference between *APOEε4* carriers and noncarriers in patients with Rett Syndrome in terms of the age of onset and clinical features. The *APOEε4* carriers exhibited the onset of regression about four months earlier with more severe symptoms than the noncarriers [238]. Recent studies showed that the recruitment of MeCP2 to a single CpG island of the *APOE* gene was noted in three different cell lines: HepG2 (hepatoma), LN-229 (glioblastoma), and SH-Sy5Y (neuroblastoma) [239]. These findings imply that the involvement of DNA methylation/MeCP2 in *APOE* transcription is critically associated with cholesterol metabolism in certain tissues, particularly in the brain.

As discussed, RTT has been proposed as a strong candidate to examine the relationship between MeCP2 and brain metabolism. Recently, a group of scientists applied an RTT mouse model B6.129P2(C)-*Mecp2^tm1.1Bird^* to examine the metabolic complex in the *Mecp2*-deficient cortex of PND50 hemizygous male mice. The study showcased that 101 metabolites that are involved in multiple metabolic pathways, including energy metabolism, lipid metabolism, amino acid metabolism, nucleotide metabolism and micronutrient metabolism, were significantly dysregulated. The researchers suggested that these dysregulated metabolites could become potential biomarkers for RTT, as clinical symptoms progressed [212]. Another study also indicated the abnormal transcript levels of HMG-CoA reductase, squalene epoxidase, and lanosterol synthase in the liver of *Mecp2* null male mice at the age of 8 weeks. These levels had increased three-fold compared to the wild type mice. Importantly, these enzymes are involved in cholesterol synthesis. The authors suggested a direct link between MeCP2 and lipid metabolism in the liver through the interactive mechanism between NCoR1, HDAC3, and MeCP2 [240]. Similarly, a study applying RNA sequencing and proteomic analysis showed an 8.1-fold increase in lanosterol synthase in the cortex of *Mecp2^Jae/y^* (*Mecp2*-deficient males) [241]. Therefore, it is conceivable that DNA methylation/MeCP2 may be greatly involved in brain metabolism.

*MECP2* mutations result in several different neurological/neurodevelopmental disorders that may have metabolic components, particularly in the brain, implying the importance of the link between MeCP2 and brain metabolism. Investigation of this association may help in finding potential treatment strategies towards MeCP2-linked neurological/neurodevelopmental disorders.

## 5. Conclusions and Implications

Although there is no overt connection between epigenetic modifications/factors, DNA methylation readers, and brain metabolism, the clinical symptoms of MeCP2-linked neurological disorders have drawn attention to metabolic defects in glucose and cholesterol metabolism, commonly observed in RTT patients. Throughout this review, we have aimed to highlight the link between DNA methylation reader(s) and brain metabolism. The discussions surrounding the role of MeCP2 in glucose and cholesterol metabolism have established that DNA methylation and MeCP2 (as a reader of DNA methylation) are vital components of mammalian neural development and RTT pathophysiology. Of these studied links, the most prominent topics include neuron and astrocyte homeostasis, neurotransmitter synthesis, moderation of autophagic activity, myelin formation, and synaptogenesis. Scientific findings have been further strengthened by mechanistic insights into MeCP2 involvement in brain physiology, epigenetic mechanisms, signaling pathways, and gene regulation at the transcription and translation levels.

The corroborated evidence obtained from studies focusing on the role of DNA methylation readers regarding MeCP2-associated neurological disorders poses immense implications on future therapeutic research. MeCP2 not only embodies a prominent candidate for investigating the mechanistic link between epigenetic changes and RTT pathophysiology, but it also signifies a greater emphasis on metabolic defects as a major side component to neurological disorders. This review ultimately sought to encourage further research into a holistic view of neurological disease progression that could include epigenetic alterations and irregular nutrient metabolism as side complications. Impact of epigenetic mechanisms/factors in unbalanced nutrient metabolism essentially underscores the need for future studies, particularly the link between epigenetic factors with glucose and cholesterol metabolism in the CNS. The resulting by-product of these proposed investigations could thus include a more robust outlook on the therapeutic potential of metabolic drugs, especially in the context of ameliorating DNA methylation reader-associated brain disorders.

## Figures and Tables

**Table 1:** MeCP2-linked neurodevelopmental disorders and metabolic impairments.

Diseases	Metabolic Impairments	References
Rett Syndrome	Abnormal systemic and brain glucose utilization as well as insulin resistance Impaired systemic and brain cholesterol metabolism Abnormal mitochondrial function	[18,19,20,21,22,23,24,25]
Autism Spectrum Disorders	Altered brain glucose utilization and insulin resistance dyslipidemia	[26,27,28]
Fetal Alcohol Spectrum Disorders	Impaired glucose tolerance Increase plasma triglyceride level	[29]
Severe Neonatal Encephalopathy	Impaired mitochondrial oxidative phosphorylation	[30,31]

## Data Availability

Not applicable.

## Abbreviations

5AZA: 5-aza-2′-deoxycytidine; 5hmC: 5-hydroxymethylcytosine; 5mC: 5-methylcytosine; Aβ: amyloid β protein; ABCA1: ATP binding cassette subfamily A member 1; ACC1: acetyl-CoA carboxylase 1; acetyl-CoA: acetyl-coenzyme A; AID: activation-induced cytidine deaminase; α-KG: α-ketoglutarate; AMPK: adenosine 5′-monophosphate activated protein kinase; APOBEC: apolipoprotein B mRNA-editing enzyme complex; ApoE: apolipoprotein E; AS160: Akt substrate of 160 kDa; ASD: autism spectrum disorder; ATP: adenosine triphosphate; ATP5B: ATP synthase F1 subunit β; BBB: blood-brain barrier; BER: base excision repair; BDNF: brain-derived neurotrophic factor; CaMKII: calcium/calmodulin-dependent protein kinase II; CpG: cytosine-phosphate-guanine di-nucleotides; COX5a: cytochrome c oxidase subunit 5a; CREB: cAMP response element binding protein; CNS: central nervous system; CTD: C-terminal domain; CYP46A1: cytochrome P450 family 46 subfamily A member 1; DNMT: DNA methyltransferase; E: embryonic day; ETC: electron transport chain; FAS: fatty acid synthase; FASD: fetal alcohol spectrum disorders; G6PD: glucose-6-phosphate dehydrogenase; G6Pase: glucose-6-phosphatase; GABA: gamma amino butyric acid; GAPDH: glyceraldehyde-3-phosphate dehydrogenase; GCM: glia-conditioned media; GFAP: glial fibrillary acidic protein; GLUTs: glucose transporters; GPS2: G protein pathway suppressor 2; H3K27: histone H3 lysine 27; HD: Huntington’s disease, HDAC: histone deacetylase; HDL: high-density lipoprotein; HIF1α: hypoxia-inducible factor 1-alpha; HMGCR: 3-hydroxy-3-methyl-glutaryl-CoA reductase; ID: intervening domain; LC3B-II: microtubule-associated protein light chain 3B-II; LDL: low-density lipoprotein; LDLr: low-density lipoprotein receptor; LTP: long-term potentiation; LXR: liver X receptor; MBD: methyl-CpG-binding domain; MBP: methyl-CpG binding protein; MDS: *MECP2* duplication syndrome; MeCP2: methyl-CpG binding protein 2 (protein); *Mecp2*: mouse gene; *MECP2*: human gene; mSIN3A: mammalian switch-independent 3A; mTOR: mechanistic target of rapamycin; NCoR: nuclear receptor corepressor; NPCs: neural progenitor cells; NPC1: Niemann-Pick C1 protein 1; NDUFB8: NADH dehydrogenase-ubiquinone 1 beta subcomplex subunit 8; NTD: N-terminal domain; PEPCK: phosphoenolpyruvate carboxykinase; PGC-1α: peroxisome-proliferator-activated receptor gamma coactivator 1 alpha; PI3K: phosphoinositide 3-kinase; PK: pyruvate kinase; PKM2: pyruvate kinase M2; PPAR: peroxisome proliferator-activated receptor; PPP: pentose phosphate pathway; PND: postnatal day; PNS: peripheral nervous system; PRAS40: proline-rich AKT substrate of 40 kDa; PTMs: post-translational modifications; PTP1B: protein tyrosine phosphatase 1B; ROS: reactive oxygen species; RGCs: retinal ganglion cells; RTT: Rett Syndrome; SAPAP3: SAP90/PSD95-associated protein 3; SCA: spinocerebellar ataxia; SLC2A3: solute carrier family 2 member 3; SMRT: silencing mediator for retinoid and thyroid hormone receptors; SRA: Set and RING-associated; SR-BI: scavenger receptor class B type I; SREBP: sterol regulatory element-binding proteins; TCA: tricarboxylic acid; TET: ten-eleven translocation; TRD: transcriptional repression domain; TRKB: tyrosine receptor kinase B; UHRF1: ubiquitin-like containing PHD and ring finger domains 1; UHRF2: ubiquitin-like containing PHD and ring finger domains 2; UTR: untranslated regions; VDAC3: voltage-dependent anion channel 3; v-SNARE: vesicle associated soluble N-ethylmaleimide-sensitive factor attachment protein receptors; ZBTB4: zinc finger and BTB domain-containing 4; ZBTB38: zinc finger and BTB domain-containing 38.
